# Luciferase mRNA Transfection of Antigen Presenting Cells Permits Sensitive Nonradioactive Measurement of Cellular and Humoral Cytotoxicity

**DOI:** 10.1155/2016/9540975

**Published:** 2016-01-05

**Authors:** Tana A. Omokoko, Uli Luxemburger, Shaheer Bardissi, Petra Simon, Magdalena Utsch, Andrea Breitkreuz, Özlem Türeci, Ugur Sahin

**Affiliations:** ^1^Division of Translational and Experimental Oncology, Department of Medicine III, Johannes Gutenberg University, Freiligrathstrasse 12, 55131 Mainz, Germany; ^2^BioNTech Cell & Gene Therapies GmbH, An der Goldgrube 12, 55131 Mainz, Germany; ^3^BioNTech AG, An der Goldgrube 12, 55131 Mainz, Germany; ^4^Ganymed Pharmaceuticals AG, An der Goldgrube 12, 55131 Mainz, Germany

## Abstract

Immunotherapy is rapidly evolving as an effective treatment option for many cancers. With the emerging fields of cancer vaccines and adoptive cell transfer therapies, there is an increasing demand for high-throughput* in vitro* cytotoxicity assays that efficiently analyze immune effector functions. The gold standard ^51^Cr-release assay is very accurate but has the major disadvantage of being radioactive. We reveal the development of a versatile and nonradioactive firefly luciferase* in vitro* transcribed (IVT) RNA-based assay. Demonstrating high efficiency, consistency, and excellent target cell viability, our optimized luciferase IVT RNA is used to transfect dividing and nondividing primary antigen presenting cells. Together with the long-lasting expression and minimal background, the direct measurement of intracellular luciferase activity of living cells allows for the monitoring of killing kinetics and displays paramount sensitivity. The ability to cotransfect the IVT RNA of the luciferase reporter and the antigen of interest into the antigen presenting cells and its simple read-out procedure render the assay high-throughput in nature. Results generated were comparable to the ^51^Cr release and further confirmed the assay's ability to measure antibody-dependent cell-mediated cytotoxicity and complement-dependent cytotoxicity. The assay's combined simplicity, practicality, and efficiency tailor it for the analysis of antigen-specific cellular and humoral effector functions during the development of novel immunotherapies.

## 1. Introduction

Cancer immunotherapy is emerging as an important contributor to the armamentarium of future oncology treatments [1–4]. This was heralded by the advent of checkpoint inhibitors, which have made a paradigm shifting difference in the outcome of cancer treatment, resulting in sustained effects and long term survival [5, 6]. Checkpoint inhibitors only unleash the effector functions of preformed T cell specificities. This has motivated the reassessment of vaccination approaches as a complementary concept [7]. As a parallel development, due to maturation of technology and promising clinical data, the interest in redirecting adoptively transferred T cells by recombinant T cell receptors (TCRs) and chimeric antigen receptors (CARs) has moved into the spotlight [8, 9], as has the pursuit of cancer-cell surface directed antibodies recruiting and activating immune effectors such as FcR positive immune cells (ADCC) or the complement cascade (CDC).

One of the many technical challenges in immunotherapy development is the assessment of cytotoxicity induced by immune effectors, whether engineered or therapeutically elicited, in biological assays. Such assays are required for different stages of immunotherapeutic product development, including but not limited to high-throughput discovery/selection of clinical lead candidates, mechanism-of-action or pharmacodynamics, biomarker studies accompanying clinical trial protocols, and potency assays for release of immunotherapeutic compounds.

Biological cytotoxicity assays for immunotherapeutic concepts may be more challenging as compared to those for chemical compounds due to various reasons. These include the use of difficult-to-label target cells, or, regarding reporter gene transfection-based assays, the use of difficult-to-transfect targets such as primary human professional antigen presenting cells (APCs). These have to be modified to efficiently express not only the reporter gene but also the antigen of interest when measuring the cytotoxicity of cytotoxic T lymphocytes (CTLs).

Many cytotoxicity assays assess the integrity of target cell membranes after coincubation with killing reagents, for example, CTLs or monoclonal antibodies (mAbs). The Chromium-51- (^51^Cr-) release assay, first described in 1968 [10], is still the gold-standard but has the drawback of being radioactive and consequently hazardous. Newer nonradioactive assays using vital dyes [11], fluorescent dyes [12, 13], and combinations thereof [14] as well as bioluminescence-based assays [15, 16] have various disadvantages ranging from suboptimal labelling of targets to spontaneous release by leaky cells and inacceptable labor intensiveness [14, 17, 18].

A commonly used nonradioactive reporter gene is the luciferase enzyme [19–21]. When expressed in living cells, luciferase produces bioluminescence through a photogenic reaction in which it catalyzes the oxygenation of luciferin taken up from a substrate buffer that is added to the wells in the presence of intracellular oxygen and ATP.

Existing plasmid-based approaches using luciferase for the assessment of cytotoxicity such as the one described by Brown et al. [22] have the drawbacks of insufficient transfection efficiencies and significant decreases in vitality when using nondividing primary cells [23].

Therefore, the objective of the project presented here was to develop an efficient nonradioactive firefly luciferase-based cytotoxicity assay system compatible with dividing and primary nondividing APCs and suitable for high-throughput screening of cytotoxicity of immunotherapeutic formats. More specifically, the assay should robustly allow the assessment of antigen-specific CTL responses, antibody-dependent cell-mediated cytotoxicity (ADCC), and complement-dependent cytotoxicity (CDC).

To this end, instead of using a plasmid-based reporter gene delivery, a gene-encoding RNA was used. RNA is a versatile format to not only deliver the nonradioactive firefly luciferase reporter into the target cells, but also allow the antigen to be recognized by the respective immune effectors. Gene-encoding RNA for engineering of cells has the advantages of being easy to produce in large amounts by* in vitro* transcription (IVT) and easy to deliver by electroporation without compromising cell viability and, since it does not need to enter the nucleus, it is also an efficient system to transfect both dividing and nondividing cells. Furthermore, this approach circumvents transcriptional regulation issues faced when using DNA plasmids [23–26].

As previously reported, we have developed a plasmid construct (pST1-*Insert*-2BglobinUTR-A120-Sap1), which upon* in vitro* transcription gives rise to a 3′ modified RNA with optimized stability and translational efficiency [27]. This is achieved by fusing the cDNA of the gene of interest to the plasmid's cassette featuring two sequential human beta-globin 3′ untranslated regions (UTRs) and a 120-nucleotide long poly(A) tail with an unmasked 3′ end.

Taking advantage of our plasmid construct, this paper presents a sensitive, rapid, and simple-to-perform luciferase IVT RNA-based bioassay applicable for high-throughput screening of cytotoxicity mediated by antigen-specific CTLs or ADCC- or CDC-inducing mAbs.

## 2. Materials and Methods

### 2.1. Cells and Cell Lines

The human erythromyeloblastoid leukaemia cell line K562 stably transfected with human HLA-A^*∗*^0201 (referred to as K562-A2) was cultured under standard conditions [28]. Endogenously human Claudin 18.2 (hCLDN18.2) expressing human gastric cancer cell lines NUGC-4 and KATO-III were maintained in RPMI 1640 (Life Technologies) supplemented with 10% foetal calf serum (Biofluid Inc., Gaithersburg, MD, USA) at 37°C, 5% CO_2_, and RPMI 1640 supplemented with 20% foetal calf serum at 37°C, 7.5% CO_2_, respectively. The CHO-K1 cell line stably expressing hCLDN18.2 was cultured in DMEM-F12 (Life Technologies), supplemented with 10% foetal calf serum, 1% Penicillin-Streptomycin (Life Technologies), and 1.5 mg/mL Geneticin (GE Healthcare Life Sciences) at 37°C, 7.5% CO_2_. Peripheral blood mononuclear cells (PBMCs) were isolated by Ficoll-Hypaque (Amersham Biosciences, Uppsala, Sweden) density gradient centrifugation from buffy coats obtained from healthy blood bank donors. Monocytes were enriched from PBMCs with anti-CD14 microbeads (Miltenyi Biotec, Bergisch-Gladbach, Germany). Immature (iDCs) and mature dendritic cells (mDCs) were generated as previously described [27]. The monospecific CTL cell line IVSB specific for the HLA-A^*∗*^0201-restricted tyrosinase-derived epitope tyr_368–376_ [29, 30] was cultured as previously described [31].

### 2.2. *In Vitro* Expansion of Human T Cells

CD8^+^ T cells were purified from PBMC of human cytomegalovirus (CMV)^+^ donors by positive magnetic cell sorting (Miltenyi Biotec) and expanded by coculturing 2 × 10^6^ effectors with 3 × 10^5^ autologous DCs either electroporated with IVT RNA or pulsed with overlapping peptide pool for 1 week in complete medium supplemented with 5% AB serum, 10 U/mL IL-2, and 5 ng/mL IL-7.

For nonspecific expansion, 2 × 10^6^ per well naïve CD8^+^ T cells purified from CMV^−^ donors were stimulated in OKT3 mAb coated 24-well plates (Janssen-Cilag GmbH, Neuss). Coating was performed using 300 *μ*L/well PBS-diluted mAb (10 *μ*g/mL) for 2 h at 37°C. After 24 h of culture, 50 U/mL IL-2 was added to the stimulated CTLs. On day 3, the cells were resuspended in fresh medium supplemented with 50 U/mL IL-2 and cultured for another 4 days in 24-well plates without OKT3.

### 2.3. Peptides and Peptide Pulsing of Stimulator Cells

Pools of N- and C-terminally free 15-mer peptides (all peptides purchased from JPT Peptide Technologies GmbH) with 11 amino acid overlaps corresponding to sequences of CMV-pp65 and HIV-gag (referred to as antigen pool), the latter used as negative control, were dissolved in DMSO to a final concentration of 0.5 mg/mL. The HLA-A^*∗*^0201 restricted peptides derived from the CMV-pp65 (pp65_495-503_, NLVPMVATV), tyrosinase (tyr_368–376_, YMDGTMSQV), and SSX2 (SSX2_41–49_, KASEKIFYV) antigens were reconstituted in PBS 10% DMSO. For pulsing, stimulator cells were incubated for 1 h at 37°C in culture medium using concentrations of 1–3 *μ*g/mL, where not otherwise indicated.

### 2.4. Vectors for* In Vitro* Transcription

A plasmid for* in vitro* transcription of the synthetic firefly luciferase reporter gene (*luc2*) was constructed based on the previously described pST1-insert-2hBgUTR-A(120) vector, which allows the generation of RNA with optimized stability and translational efficacy [27]. The* luc2* gene was subcloned from the pGL4.14[luc2/Hygro] vector (Promega Corporation, Madison, WI, USA) and an internal EciI restriction site deleted by site-directed mutagenesis (Agilent) using the oligo luc2mut sense (5′-CTA CCA GGC ATC CG **A** CAG GGC TAC GGC CTG ACA GAA AC-3′) and the reverse complement (Eurofins Genomics).

The pST1-2hBgUTR-A(120)-IVT vectors containing enhanced green fluorescent protein (eGFP) and the full-length TCR alpha and beta chains of the pp65_495–503_-specific and HLA-A^*∗*^0201-restricted TCR-8-CMV#14 have been previously described [27, 31]. The pp65 antigen-encoding vector pST1-sec-pp65-MITD-2hBgUTR-A(120) features a signal sequence for routing to the endoplasmic reticulum and the MHC class I transmembrane and cytoplasmic domains to improve MHC class I and II presentation [32].

### 2.5. Generation of IVT RNA and Transfer into Cells

IVT RNA was generated as previously described [27] and added to cells suspended in X-VIVO 15 medium (Lonza) in a precooled 4 mm gap sterile electroporation cuvette (Bio-Rad). Electroporation was performed with a Gene-Pulser-II apparatus (Bio-Rad; human iDC: 276 V/150 *μ*F; human mDC: 290 V/150 *μ*F; CD8^+^ T cells: 450 V/250 *μ*F; K562-A2, CHO and NUGC-4: 200 V/300 *μ*F; KATO III: 250 V/475 *μ*F).

### 2.6. Flow Cytometric Analysis

Flow cytometric analysis was performed on a FACS-Calibur analytical flow cytometer using CellQuest-Pro software (BD Biosciences). DC maturation markers were detected by staining with PE-labelled anti-CD83 and APC-labelled anti-HLA-DR antibodies (BD Biosciences).

### 2.7. Luciferase-Based CTL Cytotoxicity Assay

APCs were electroporated with 10–50 *μ*g of* luc2* IVT RNA. For coelectroporation experiments,* luc2* IVT RNA and either pp65 or control RNA were electroporated into the target cells simultaneously. After electroporation, cells were resuspended in prewarmed culture medium and incubated overnight at 37°C and 5% CO_2_. 20 h later, cells were diluted to a final concentration of 2 × 10^6^ cells/mL in culture medium containing 1–3 *μ*g/mL specific peptide (pool) or control peptide (pool) and were incubated for 1 h at 37°C and 5% CO_2_. After pulsing with peptides, cells were washed and resuspended in complete culture medium and 1 × 10^4^ cells per well were plated in triplicate in 50 *μ*L into white 96-well flat-bottom plates (Thermo Scientific). CD8^+^ effector cells were washed, counted, and cocultured in different E : T ratios in a final volume of 100 *μ*L per well at 37°C and 5% CO_2_ for 3 h. Minimal and maximal lysis control wells contained 1 × 10^4^ target cells alone in a total volume of 100 *μ*L and 90 *μ*L, respectively. After the specified time 50 *μ*L of a D-luciferin substrate solution containing 3.6 mg/mL D-luciferin (BD Biosciences Pharmingen), 150 mM HEPES (Life Technologies) and 1.2 mU/*μ*L Adenosine 5′-Triphosphatase (Sigma-Aldrich) were added to each well to a final volume of 150 *μ*L. Maximum lysis control wells were treated with 10 *μ*L 2% Triton X-100/PBS prior to addition of substrate. 96-well plates were incubated for another hour at 37°C and 5% CO_2_. After a total coincubation time of 4 h, the intracellular luciferase activity of living cells was measured using a Tecan Infinite M200 reader (Tecan Group AG, Crailsheim, Germany). Percent specific lysis was calculated as follows:

(1) $$\begin{array}{c} \left(\;1-\frac{{\text{CPS}}_{\text{experimental}}-{\text{CPS}}_{\text{minimal}}}{{\text{CPS}}_{\text{maximal}}-{\text{CPS}}_{\text{minimal}}}\;\right)\times 100. \end{array}$$

### 2.8. ^51^Cr-Release Assay

Autologous DCs were loaded with 3 *μ*g/mL pp65 peptide pool or control peptide and labelled with 100 *μ*Ci of ^51^Cr (NEN Life Science) for 90 min at 37°C and 5% CO_2_. ^51^Cr labelled DCs were washed and resuspended in complete culture medium and 1 × 10^4^ targets per 200 *μ*L per well coincubated in triplicate with effector T cells at different E : T ratios for 4 h. A total of 60 *μ*L of the supernatant was harvested, and released ^51^Cr was measured with a scintillation counter. Spontaneous release was also determined. Percent specific lysis was calculated using the following equation:

(2) $$\begin{array}{c} \frac{\text{experimental release}-\text{spontaneous release}}{\text{maximum release}-\text{spontaneous release}}\times 100. \end{array}$$

### 2.9. Luciferase-Based ADCC Assay

Target cells were electroporated using 7 *μ*g of* luc2* IVT RNA. After electroporation, cells were resuspended in 2.4 mL prewarmed culture medium. 2 × 10^4^ KATO-III cells or 2.5 × 10^4^ NUGC-4 cells per 50 *μ*L per well were plated independently in triplicate into white 96-well flat-bottom plates (NUNC) and were incubated for 4–6 h at 37°C, 7.5%, and 5% CO_2_, respectively. Different IMAB 362 concentrations ranging from 0.06 ng/mL to 200 *μ*g/mL and Ficoll-Paque-purified PBMCs from healthy donors were added to each well (E : T ratio of 40 : 1). KATO-III and NUGC-4 cell-containing plates were incubated for 24 h at 37°C, 7.5%, and 5% CO_2_, respectively. After overnight incubation, 10 *μ*L 8% Triton X-100/PBS solution was added to the maximum lysis control wells and 10 *μ*L PBS to the other wells. Finally, 50 *μ*L D-luciferin substrate solution containing 3.2 mg/mL D-luciferin, 160 mM HEPES, and 0.64 mU/*μ*L Adenosine 5′-Triphosphatase was added to each well to a final volume of 160 *μ*L and plates were incubated for 90 min at room temperature (RT) in the dark. Bioluminescence was measured using a luminometer (Infinite M200, TECAN). Percentage of specific lysis was calculated using the formula described above for the luciferase-based CTL cytotoxicity assay.

### 2.10. Luciferase-Based CDC Assay

CHO-K1~hCLDN18.2 cells were electroporated using 7 *μ*g of* luc2* IVT RNA and resuspended in 2.4 mL prewarmed culture medium. 5 × 10^4^ cells per 50 *μ*L per well were plated in triplicate into white 96-well flat-bottom plates (NUNC) and were incubated for 24 h at 37°C, 7.5% CO_2_. 44% human serum from healthy donors was prepared in RPMI medium supplemented with 20 mM HEPES. IMAB 362 antibody was diluted in human serum to final assay concentrations ranging from 31.6 ng/mL to 10 *μ*g/mL. 50 *μ*L of different IMAB 362 antibody concentrations were added to the target cells to achieve an end concentration of 20% (v/v) serum. The 96-well plate was incubated for 80 min at 37°C, 7.5% CO_2_. After incubation, 10 *μ*L 8% Triton X-100/PBS solution was added to control for maximum lysis and 10 *μ*L PBS to the remaining wells. D-Luciferin substrate solution was added to each well as described above for the ADCC assay. Plates were incubated for 45 min at RT in the dark and then measured in a luminometer (Infinite M200, TECAN). Specific lysis was calculated as described above for the ADCC assay.

## 3. Results and Discussion

### 3.1. Electroporation of Firefly Luciferase IVT RNA into Dividing and Nondividing APCs Is Nontoxic and Leads to Strong and Long-Lasting Gene Expression

One of the key elements for the performance of a cytotoxicity assay system is the labelling of the target cell population with a reporter system without affecting cell viability. This limitation is pronounced when using nondividing cells, such as primary human APCs, frequently required in the context of immunotherapy drug development. Plasmid-based reporter gene assays are of low efficiency and do not provide a good solution [22]. Instead of using a plasmid-based delivery approach, the use of a luciferase gene-encoding mRNA was investigated here.

**Figure 1:**
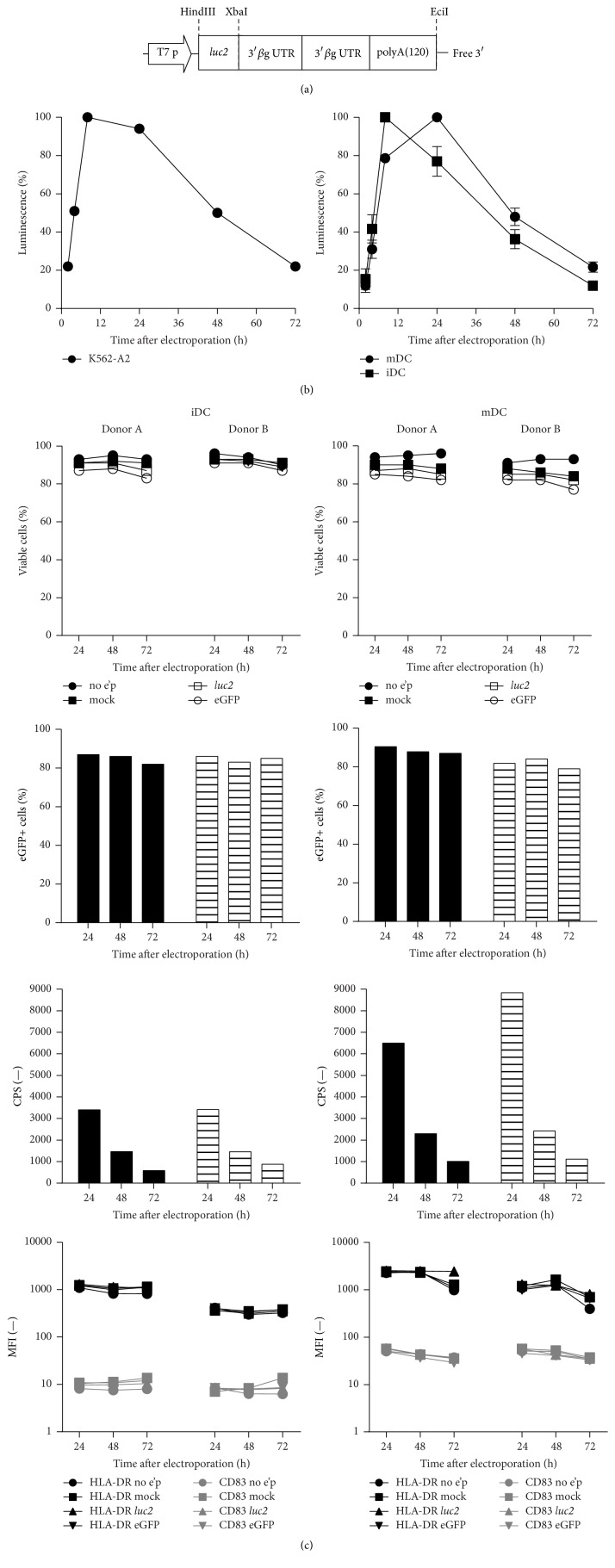
Electroporation of firefly luciferase IVT RNA into DCs and K562-A2 cells is nontoxic and leads to strong and long-lasting gene expression without affecting target cell phenotype. (a) Optimised* luc2* reporter vector: composed of a gene-optimized synthetic firefly luciferase reporter gene cloned in front of two human *β*-globin 3′ untranslated regions (UTRs) fused head to tail and an unmasked free poly(A) tail of 120 bp. (b) Kinetics of* luc2* expression in K562-A2 cells (*n* = 1), human iDCs (*n* = 3), and mDCs (*n* = 3). Cells transfected with 8 pmol of* luc2*-encoding IVT RNA were harvested at different time points to measure luminescence from 1 × 10^4^ cells (Bright-Glo Luciferase Assay Kit for 96-well plates (Promega)). Results are the mean ± SD luminescence. Percent luminescence is relative to the highest luminescence signal obtained in each experiment. (c) Viability, reporter gene expression of iDCs and mDCs after eGFP and* luc2* electroporation and phenotype after electroporation are depicted in descending order, respectively. iDCs (left panel) and mDCs (right panel) of 2 different donors were transfected with 10 *μ*g eGFP- or* luc2*-encoding IVT RNA. Negative controls: cells electroporated without RNA (mock) and unelectroporated (no e'p) cells. Cells were harvested at different time points. Viability and HLA-DR, CD83, and eGFP expression levels were determined by flow cytometry. Luciferase activity of 1 × 10^4^ viable cells was measured by luminescence in triplicate.

The firefly luciferase (*luc2*) gene was cloned into the pST1-2hBgUTR-A(120)-EciI vector and* in vitro* transcribed from this construct with a stability optimized 3′ end (Figure 1(a)). 5.4 *μ*g of this IVT RNA was electroporated into K562 leukemic cells stably transfected with the human HLA-A^*∗*^0201 gene (hereinafter referred to as K562-A2) [28]. In addition, difficult-to-transfect nondividing primary human cells, namely, iDCs and mDCs, were also used as targets (Figure 1(b)). Luminescence after D-luciferin substrate addition was instantly detected and strongly increased between 2 and 8 h after electroporation. Signals reached maximum levels after 10 to 24 h in all cell types. K562-A2 cells showed the highest signal levels. Activity in primary cells was also very robust, and a 2–4-fold higher maximum luciferase activity was detected in mDCs compared to iDCs. High and durable expression levels were achieved with an approximately 80% signal intensity still being detectable 36 h after electroporation into K562-A2 cells and mDCs, and 24 h in the case of iDCs. Luciferase expression kinetics exhibited batch consistency within each cell type and were not affected by the use of higher* luc2* IVT RNA amounts (data not shown).

To assess the viability of the target cells, 10 *μ*g RNA encoding luciferase and eGFP were electroporated into human iDCs and mDCs generated from the same donor. Both iDCs and mDCs displayed excellent viability, ranging from 85 to 95% in the 72 h after electroporation with reporter RNA as determined by flow cytometry (Figure 1(c)). 80–90% of all living DCs expressed eGFP stably over 72 h illustrating high transfection efficiency. This gives the IVT RNA approach an advantage over plasmid based assays, which show low efficiencies when used with nondividing primary APCs, probably a consequence of using more stringent electrical settings [23]. Both iDCs and mDCs retained their phenotypes after electroporation as demonstrated by the sustained levels of the maturation markers HLA-DR and CD83 in* luc2* transfected cells compared to controls for as long as 72 hours after electroporation. As expected, mDCs showed a higher expression of both markers (Figure 1(c)).

In summary, the data demonstrate high, stable, and long-lasting expression of* luc2* reporter IVT RNA in dividing as well as nondividing primary APCs, without compromising the viability or immunological phenotype of the target cells.

### 3.2. Optimization of the Assay Parameters Enhances and Prolongs Luciferase Signals Whilst Minimizing Background and Reveals a Strict Luminescence to Cell Number Correlation

As a next step, the implementation of the IVT RNA-based reporter-gene engineering of target cells into a robust cytotoxicity assay with a favorable signal-to-noise ratio and a high sensitivity was investigated.

**Figure 2:**
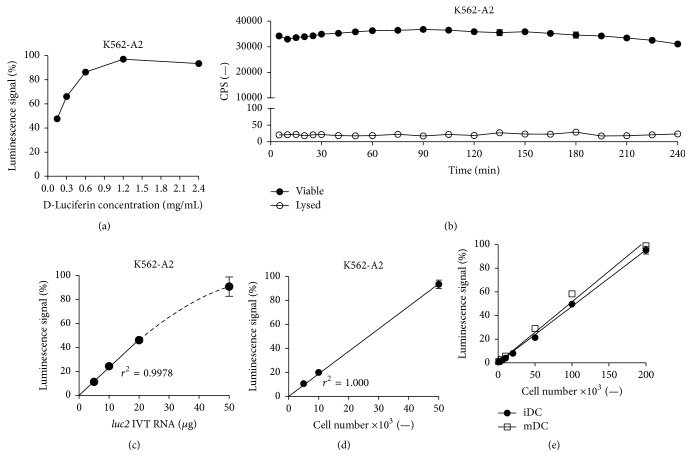
Optimization of the assay parameters enhances and prolongs luciferase signals whilst minimizing background reading and reveals a strict luminescence to cell number correlation. (a) Optimal D-luciferin substrate concentration. 1 × 10^6^ K562-A2 cells were transfected with 20 *μ*g* luc2* IVT RNA. After 24 h, luminescence of 5 × 10^4^ cells per well was measured following addition of D-luciferin in different concentrations. (b) Stable bioluminescence upon D-luciferin substrate addition and immediate abolition of signals following total cell lysis. 2.5 × 10^6^ K562-A2 cells were transfected with 50 *μ*g* luc2* IVT RNA. 24 h after transfection, luminescence of 5 × 10^4^ viable or 0.2% Triton X-100 treated cells was repeatedly measured after a single administration of 1.2 mg/mL D-luciferin substrate. Graph represents mean ± SD luminescence (*n* = 3). (c) Luminescence is dependent on* luc2* IVT RNA dose. 1 × 10^6^ K562-A2 cells were transfected with different amounts of* luc2* IVT RNA. 24 h after transfection, luminescence of 5 × 10^4^ cells per well was measured. (d) Luminescence is linearly dependent on the number of transfected K562-A2 cells. 1 × 10^6^ K562-A2 cells were transfected with 50 *μ*g* luc2* IVT RNA. 24 h after transfection, 1.2 mg/mL D-luciferin was added and luminescence of different amounts of cells was measured. (e) Luminescence is linearly dependent on the number of transfected primary cells. Human iDCs and mDCs were electroporated with 50 *μ*g* luc2* IVT RNA. 24 h after transfection, 1.2 mg/mL D-luciferin was added and luminescence of different amount of cells was measured. Cell number and signal intensity correlation (*p* < 0.0001, *r* ^2^ = 0.9984 (iDC) and 0.9923 (mDC)). Graphs (a), (c), (d), and (e) represent mean ± SD luminescence (*n* = 3) relative to the highest luminescence signal obtained within each experiment.

For K562-A2 cells electroporated with 20 *μ*g of* luc2* IVT RNA, a D-luciferin substrate concentration of 1.2 mg/mL achieved the highest signals (Figure 2(a)). These signals were prolonged and stable, allowing continuous detection of living cells after a single administration of substrate for at least 4 h (Figure 2(b)). Bioluminescence dropped to levels close to zero following Triton X-100 detergent-mediated cell lysis, demonstrating responsiveness of the technique to cytotoxic events (Figure 2(b)). The rapid reduction of background signals from dying cells is further accelerated by the addition of ATPase to the substrate buffer, which results in the immediate hydrolysis of ATP released from these cells and ceasing of luciferase activity following cell death (data not shown). The stability of the signal over 4 hours and the direct assessment of cell death allow both endpoint measurements and the determination of killing kinetics, which is superior to many other assays such as the ^51^Cr and the Europium release assays, that only allow the former [17, 18, 33].

Electroporation of K562-A2 cells with increasing amounts of* luc2* IVT RNA displayed a dose-dependent increase in luminescence (Figure 2(c)).

The strict linear dependence between the detectable bioluminescence and transfected cell numbers further verified the sensitivity of the method (Figure 2(d)).

Next, these conditions were tested on nondividing primary cells, namely, human monocyte-derived iDCs and mDCs. Addition of D-luciferin to human iDCs and mDCs 24 h after their electroporation with* luc2* IVT RNA also demonstrated a linear correlation between cell number and bioluminescence (Figure 2(e)). Luciferase activity from as few as 1,000 cells was more than 24-fold higher than background levels, implying that luminescence from such few cells suffices for accurate reporter gene detection (Figure 2(e)).

The equipment and the read-out conditions of the assay greatly affect the specific signal, background reading, and the cross-talk between wells. In our hands, white polystyrene flat-bottom plates that reflect light and maximize the output signal and the more cost-efficient Tecan Infinite M200 luminescence plate reader (Tecan, Crailsheim, Germany) resulted in an excellent signal-to-noise ratio, achieving specific signals with multiple logs above background (Table 1; Figure 2(b)). That, along with the advancements in plate readers, such as the automatic regulation of temperature and reagent addition, further promotes the automation of this assay for high-throughput screening. It should be noted that one can easily modify the assay according to one's needs, for example, target cell type and amount of cells usually available, by choosing a suitable plate reader and adjusting the amount of luciferase IVT RNA used for electroporation.

### 3.3. Luciferase IVT RNA Electroporation Permits Assessment of Antigen-Specific CTL Activity Comparable to the ^51^Cr Release and Superior in the Ability to Monitor Killing Kinetics

**Figure 3:**
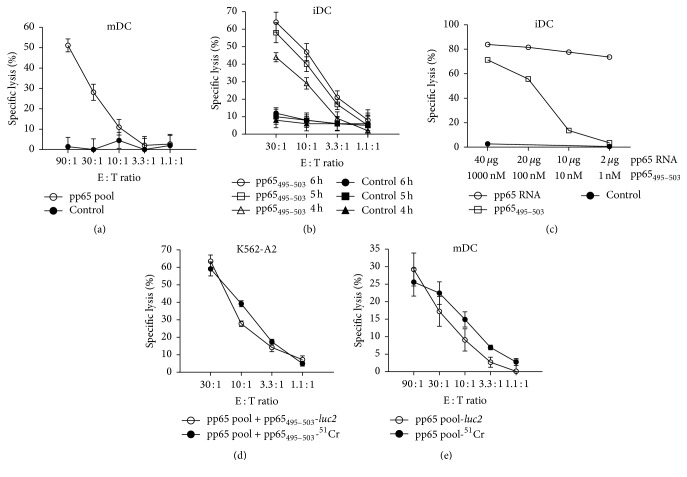
Luciferase IVT RNA electroporation permits assessment of antigen-specific CTL activity comparable to the ^51^Cr release and superior in the ability to monitor killing kinetics. (a) Cytolytic activity of primary CMV-pp65-specific T cells. CMV^+^ donor-derived CD8^+^ T cells were expanded for one week and used to assess the killing of autologous mDCs transfected with 50 *μ*g* luc2* RNA and loaded with overlapping peptide pools representing either CMV-pp65 or HIV-gag as control. Specific lysis was determined after 4 h incubation of peptide-loaded target cells with CD8^+^ effector cells using different E : T ratios. (b) Kinetics of killing mediated by TCR-transfected CD8^+^ T cells. OKT3-stimulated CD8^+^ T cells of a CMV^−^  HLA-A^*∗*^0201^+^ donor were transfected with 20 *μ*g TCR-8-CMV#14 alpha and beta chain IVT RNAs. Autologous iDCs transfected with 20 *μ*g* luc2* RNA were loaded with either peptide pp65_495–503_ or tyr_368–376_ as control. iDCs and CD8^+^ T cells were cocultured at different E : T ratios and specific killing was assessed at different time points. (c) Dose-dependent killing of target cells using different antigen formats. OKT3-stimulated CD8^+^ T cells from a CMV^−^  HLA-A^*∗*^0201^+^ donor were electroporated with 20 *μ*g TCR-8-CMV-#14 IVT RNA. Autologous iDCs were cotransfected with 20 *μ*g* luc2* IVT RNA and decreasing amounts of a CMV-pp65 antigen-encoding IVT RNA or were* luc2* transfected and subsequently pulsed with titrated amounts of peptide pp65_495–503_. iDC transfected with* luc2* IVT RNA and pulsed with 1000 nM SSX2_41–49_ peptide served as a control. Effector and target cells were incubated at an E : T ratio of 19 : 1. ((d) and (e)) Comparability of the* luc2* IVT RNA assay with the ^51^Cr assay. Cytotoxicity of CMV-pp65-specific CD8^+^ T cells of a HLA-A^*∗*^0201^+^ CMV^+^ donor against (d) K562-A2 cells or (e) autologous mDCs was assessed after one week antigen-specific expansion using the* luc2* IVT RNA assay in comparison to the ^51^Cr assay. 20 h after* luc2* RNA electroporation, target cells were loaded with pp65_495–503_ peptide either alone or together with 100 *μ*Ci of ^51^Cr. 1 × 10^4^ peptide-loaded targets were incubated at different E : T ratios with CD8^+^ effector cells for 4 h. Cytotoxicity was determined via measurement of luminescence after addition of D-luciferin substrate or via measurement of released ^51^Cr after harvesting of supernatant. All graphs represent the mean ± SD lysis (*n* = 3).

Having optimized the key performance parameters of the assay system, the CMV-pp65 model antigen was used to measure primary antigen-specific CTL responses, as is frequently required in vaccine approaches. To generate the respective reagents, effector T cells from a CMV^+^ donor were expanded by coculture with pp65 antigen pool loaded autologous iDCs. Simultaneously, mDCs of the donor were generated and electroporated with* luc2* IVT RNA. 20 h after transfection, the autologous target cells were loaded with either pp65 antigen pool or control antigen pool before being cocultured with the CD8^+^ effectors at different E : T ratios for 4 h. The calculated specific lysis of pp65 pulsed target cells increased with increasing E : T ratios, while target cells pulsed with control peptides were not lysed, illustrating the assays ability to detect and quantify antigen-specific CTL immune responses (Figure 3(a)).

In order to assess the assay's capacity of directly determining the kinetics of CTL-mediated killing, OKT3-stimulated CD8^+^ T cells from a CMV^−^  HLA-A^*∗*^0201^+^ donor were electroporated with IVT RNA encoding a previously isolated T cell receptor (TCR-8-CMV-#14) directed against the immunodominant CMV-pp65-derived HLA-A^*∗*^0201-restricted peptide pp65_495–503_ [31]. Autologous iDCs were transfected with* luc2* IVT RNA and 20 h later loaded with either the specific or a control peptide. Effector and target cells were incubated at different E : T ratios. D-Luciferin was added once after 3 h. Following that, multiple luminescence readouts were taken at different time points and descriptive killing kinetics could be recorded (Figure 3(b)). For each E : T ratio, the specific lysis increased over time, with the 30 : 1 ratio showing the highest specific lysis at all time points, while control peptide loaded iDCs were not lysed.

Other popular flow cytometry based cytotoxicity assays monitor, for example, caspase activation or granzyme B substrate cleavage in target cells [34]. These alternatives have the ability to quantify target cell death at the single-cell level. However, one needs to carefully determine the best time for such endpoint measurements, as markers of apoptosis such as caspase activity are only transiently present. This may be challenging especially with regard to T cell populations with unknown or low frequency antigen-specific effectors. Due to the long-lasting signals, the luciferase assay, on the other hand, provides the opportunity to take multiple measurements over a longer time period.

A further advantage of using gene-encoding RNA is that, together with the luciferase reporter gene IVT RNA, any other antigen (or vaccine) IVT RNA of interest can be cotransfected. OKT3-stimulated CD8^+^ T cells from a CMV^−^  HLA-A^*∗*^0201^+^ donor were electroporated with the same TCR-8-CMV-#14 IVT RNA. Autologous iDCs were cotransfected with* luc2* IVT RNA and decreasing amounts of a pp65 antigen-encoding IVT RNA or were* luc2* transfected and subsequently pulsed with titrated amounts of the pp65_495–503_ peptide. In addition to illustrating the efficient cotransfection of luciferase and varying amounts of antigen IVT RNA, the results show the sensitive recognition of antigen via the TCR with 74% specific lysis being detected using 2 *μ*g of pp65 RNA for transfection (Figure 3(c)). In the context of this cotransfection ability, it should be noted that the use of a full-length antigen-encoding IVT RNA would allow the detection of CTL responses specific for naturally processed epitopes that are presented on the surface of the APCs.

The ^51^Cr-release assay is widely used and is considered as the gold standard approach to assess T cell and natural killer cell-mediated cytotoxicity [32, 35–37]. The efficiency of the luciferase IVT RNA assay was thus further confirmed by a direct comparison with the ^51^Cr-release assay using either K562-A2 cells or primary DCs as target cells. For the former, which were stably transfected with HLA-A^*∗*^0201 (Figure 3(d)), effector T cells of a CMV^+^  HLA-A^*∗*^0201^+^ donor were expanded using peptide loaded autologous iDCs. K562-A2 cells were electroporated with* luc2* IVT RNA. 20 h later, half of the cells were loaded with pp65 antigen pool and pp65_495–503_ peptide alone and the other half were simultaneously labelled with ^51^Cr. For the primary DCs (Figure 3(e)), effector T cells from a CMV^+^ donor were expanded using peptide loaded autologous iDCs. In parallel, autologous mDCs were electroporated with* luc2* IVT RNA. 20 h later, half of the cells were loaded with pp65 antigen pool or control antigen pool alone and the other half were concurrently labelled with ^51^Cr. In both settings, peptide-loaded targets and CD8^+^ effector cells were then incubated at different E : T ratios for 4 h before luminescence and released chromium were measured. The IVT RNA-based assay yielded specific lysis levels that were as sensitive as, and almost identical to, the ^51^Cr assay, with 60% and ~20% specific lysis of the K562-A2 cells (Figure 3(d)) and autologous mDCs (Figure 3(e)), respectively, at an E : T ratio of 30 : 1.

### 3.4. Luciferase IVT RNA Electroporation Permits a Highly Sensitive Assessment of Antigen-Specific CTL Activity

**Figure 4:**
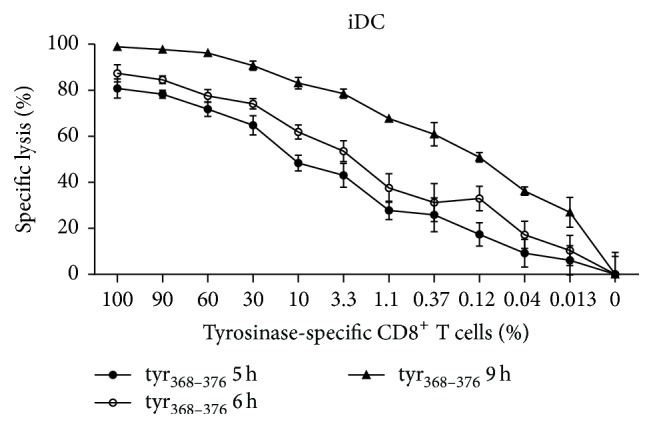
Luciferase IVT RNA electroporation permits a highly sensitive assessment of antigen-specific CTL activity. Titrated amounts of IVSB cells were spiked into PBLs and the specific lysis of autologous iDCs pulsed with the tyr_368–376_ peptide was assessed using an E : T ratio of 20 : 1. Luciferase signals were analysed after increasing coincubation times. Results are the mean ± SD (*n* = 3).

Having proven the robustness of the system, the capability of the assay to detect low-frequency antigen-specific T cells was examined. The monospecific CTL cell line IVSB recognizing the HLA-A^*∗*^0201-restricted tyrosinase-derived epitope tyr_368–376_ was used [29, 30]. Decreasing amounts of IVSB T cells were spiked into peripheral blood lymphocytes (PBLs). The specific lysis of autologous iDCs pulsed with the tyr_368–376_ peptide was assessed. Since specific lysis is calculated using internal maximum and minimum references (see Section 2), iDCs plus PBLs without IVSB T cells were used as the minimum lysis reference in this experiment. Luciferase signals were analyzed after 5, 6, and 9 h of coincubation (Figure 4). After 5 h, the cytotoxic activity of 0.37% antigen-specific T cells corresponding to 740 IVSB cells in a total of 200,000 PBLs was easily detected based on the specific lysis of tyr_368–376_ peptide pulsed target cells (Figure 4). When the incubation time was prolonged to 9 h, the detection limit was improved to as few as 26 antigen-specific T cells, corresponding to a frequency of 0.013% of PBLs.

The results confirm the suitability of the developed assay to sensitively detect cytotoxicity induced by very rare antigen-specific T cells, as is the case with* ex vivo* tumor-antigen specific effector cells in the blood of cancer patients.

In summary, the data indicates that the luciferase IVT RNA assay performs at least as well as the ^51^Cr assay and is superior in its sensitivity, nonradioactivity, easy read-out procedure, and the monitoring of killing kinetics.

### 3.5. The Luciferase IVT RNA-Based Assay Efficiently Assesses mAb-Induced ADCC and CDC of Tumor Cell Lines

**Figure 5:**
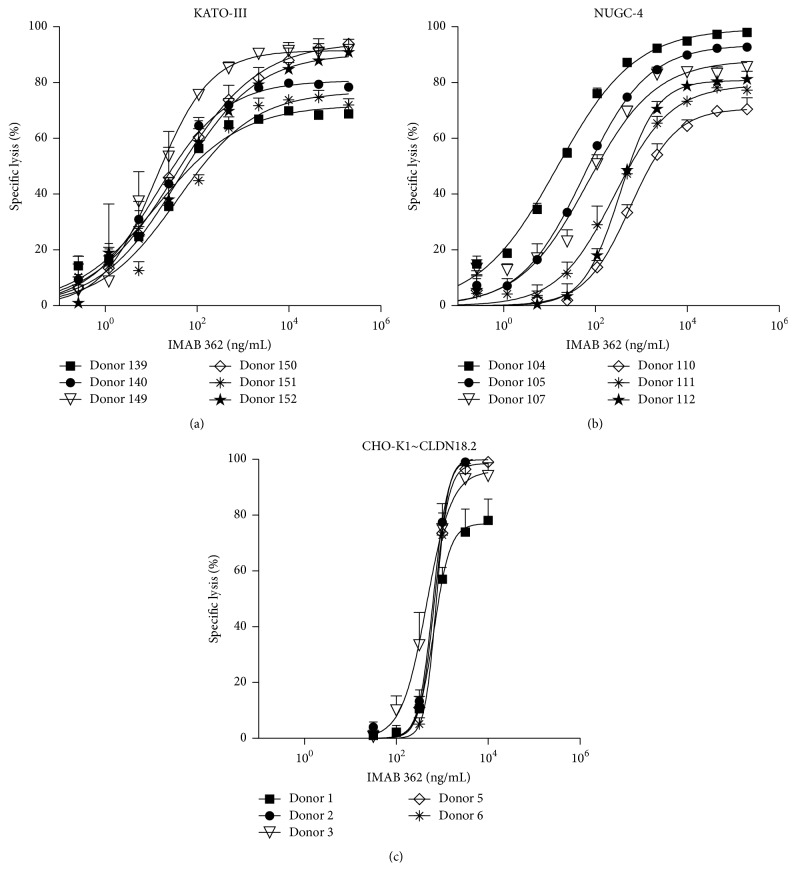
The luciferase IVT RNA-based assay efficiently assesses mAb-induced ADCC and CDC of tumour cell lines. ADCC assay using (a) KATO-III and (b) NUGC-4 cells. KATO-III and NUGC-4 cells endogenously expressing hCLDN18.2 were transfected with 7 *μ*g* luc2* IVT RNA and seeded into 96-well plates independently. 4 h later, IMAB 362 at different concentrations and human PBMCs (E : T ratio = 40 : 1) from 6 different donors were added to the target cells and incubated for 24 h. ADCC was determined 40 and 45 min after addition of D-luciferin substrate to the KATO-III and NUGC-4 cells, respectively. (c) CDC assay. CHO-K1 cells stably expressing hCLDN18.2 were transfected with 7 *μ*g* luc2* IVT RNA and seeded into 96-well plates. 24 h later, cells were incubated for 80 min with IMAB 362 diluted in human serum (final concentration of 20%) from 6 different healthy donors. CDC was determined 45 min after addition of D-luciferin substrate. Results are the mean ± SD (*n* = 3).

In addition to T cell-mediated cytotoxicity, other effector functions have also been shown to participate in antitumor responses [38]. The assay was therefore adopted to assess ADCC and CDC, which are mediated by the recruitment and activation of either FcR positive effector cells or complement factors by the Fc domains of cell-bound mAbs [39]. To this end, IMAB 362, a therapeutic mAb in advanced clinical development directed against the pan-cancer cell surface antigen Claudin 18.2 (CLDN18.2), which exerts tumor cell death via ADCC and CDC, was used [40–43]. KATO-III and NUGC-4 tumor cells endogenously expressing CLDN18.2 or CHO-K1 cells stably expressing the antigen were electroporated with* luc2* IVT RNA. To measure ADCC, IMAB 362 was added to the KATO-III (Figure 5(a)) and NUGC-4 (Figure 5(b)) target cells 4–6 h after* luc2* electroporation. The cells were then incubated with human PBMCs at a 40 : 1 E : T ratio for 24 h; then D-luciferin substrate was added for luminescence measurement. For the assessment of CDC, 24 h after* luc2* electroporation, the CHO-K1 cells were incubated with IMAB 362, diluted in human serum, for 80 minutes as a source of complement factors. Thereafter, D-luciferin was added for the signal read-out (Figure 5(c)).

Specific lysis via ADCC and CDC was found to be dependent on the IMAB 362 concentration. Dose-response curves were sigmoid with a good dynamic range. For IMAB 362-induced ADCC-mediated specific lysis of KATO-III cells, as few as 1.19 ng/mL antibody was sufficient to induce 25% killing (Figure 5(a)). For NUGC-4 cells, 24 ng/mL antibody induced 14 to 76% ADCC-mediated lysis among the different donors (Figure 5(b)). The maximum specific cell lysis was approximately 80% in both cell lines and was reached at concentrations of 9.88 *μ*g/mL IMAB 362. Robust CDC was measured at a concentration of 1000 ng/mL and reached a maximum of up to 99% lysis of CHO-K1 cells at an IMAB 362 concentration of 3.16 *μ*g/mL (Figure 5(c)).

The data demonstrates that the luciferase IVT RNA cytotoxicity assay may be used for both ADCC and CDC assessment. This may be very useful for high-throughput testing in the discovery and selection process of therapeutic mAb candidates as well as the assessment of immune cell and humoral responses in clinical vaccine development [44, 45].

## 4. Conclusions

This paper reports the establishment of a highly suitable nonradioactive IVT RNA firefly luciferase-based cytotoxicity assay. By directly measuring intracellular luciferase activity, the assay efficiently assesses effector cell cytotoxicity mediated by antigen-specific CTLs when using cell lines and primary nondividing APCs as targets. The results generated were comparable to the gold-standard ^51^Cr-release assay. Taking advantage of an optimized IVT RNA reporter, the approach is extremely sensitive and rapid and has a simple read-out procedure, rendering it applicable for high-throughput screening. In further support of this, the assay allows for the cotransfection of luciferase and the antigen-encoding RNA into the APCs followed by the subsequent monitoring of killing kinetics. The assay was adopted for the evaluation of ADCC and CDC by a cancer cell surface antigen directed mAb. Together, the properties of the developed assay render it an attractive approach for measuring cytotoxicity* in vitro*, tailored for the use in the rapidly advancing tumor vaccine development, tumor-specific TCR, and mAb discovery fields.

## Figures and Tables

**Table 1:** White opaque flat-bottom plates together with the Infinite M200 (Tecan) plate reader result in a robust signal-to-noise ratio.

Parameter	Experiment	Outcome
Plate opacity	White opaque versus transparent	Specific luciferase signals obtained from white opaque plates were 2-3-fold higher

Plate design	Flat-bottom versus V-shaped bottom	Only negligible well-to-well cross-talk was observed when flat-bottom plates were used

Plate reader	Wallac VICTOR2 (Perkin Elmer) versus Infinite M200 (Tecan) versus GENios Pro (Tecan)	Signals from the Infinite M200 device were 4-fold and signals from the GENios Pro 20-fold higher than those detected with the Wallac VICTOR2

## References

[1] Wolchok J. D., Chan T. A. (2014). Cancer: antitumour immunity gets a boost. *Nature*.

[2] Phimister E. G., Melief C. J. (2015). Mutation-specific T cells for immunotherapy of gliomas. *The New England Journal of Medicine*.

[3] Sznol M., Longo D. L. (2015). Release the hounds! Activating the T-cell response to cancer. *The New England Journal of Medicine*.

[4] Kreiter S., Vormehr M., van de Roemer N. (2015). Mutant MHC class II epitopes drive therapeutic immune responses to cancer. *Nature*.

[5] Eggermont A. M., Maio M., Robert C. (2015). Immune checkpoint inhibitors in melanoma provide the cornerstones for curative therapies. *Seminars in Oncology*.

[6] Sharma P., Allison J. P. (2015). The future of immune checkpoint therapy. *Science*.

[7] Le D. T., Lutz E., Uram J. N. (2013). Evaluation of ipilimumab in combination with allogeneic pancreatic tumor cells transfected with a GM-CSF gene in previously treated pancreatic cancer. *Journal of Immunotherapy*.

[8] Rosenberg S. A., Restifo N. P. (2015). Adoptive cell transfer as personalized immunotherapy for human cancer. *Science*.

[9] Omokoko T., Simon P., Türeci Ö., Sahin U. (2015). Retrieval of functional TCRs from single antigen-specific T cells: toward individualized TCR-engineered therapies. *OncoImmunology*.

[10] Brunner K. T., Mauel J., Cerottini J. C., Chapuis B. (1968). Quantitative assay of the lytic action of immune lymphoid cells on 51-Cr-labelled allogeneic target cells in vitro; inhibition by isoantibody and by drugs. *Immunology*.

[11] Heo D. S., Park J.-G., Hata K., Day R., Herberman R. B., Whiteside T. L. (1990). Evaluation of tetrazolium-based semiautomatic colorimetric assay for measurement of human antitumor cytotoxicity. *Cancer Research*.

[12] Korzeniewski C., Callewaert D. M. (1983). An enzyme-release assay for natural cytotoxicity. *Journal of Immunological Methods*.

[13] Blomberg K., Granberg C., Hemmilä I., Lövgren T. (1986). Europium-labelled target cells in an assay of natural killer cell activity. I. A novel non-radioactive method based on time-resolved fluorescence. *Journal of Immunological Methods*.

[14] Lichtenfels R., Biddison W. E., Schulz H., Vogt A. B., Martin R. (1994). CARE-LASS (calcein-release-assay), an improved fluorescence-based test system to measure cytotoxic T lymphocyte activity. *Journal of Immunological Methods*.

[15] Crouch S. P. M., Kozlowski R., Slater K. J., Fletcher J. (1993). The use of ATP bioluminescence as a measure of cell proliferation and cytotoxicity. *Journal of Immunological Methods*.

[16] Karimi M. A., Lee E., Bachmann M. H. (2014). Measuring cytotoxicity by bioluminescence imaging outperforms the standard chromium-51 release assay. *PLoS ONE*.

[17] Schäfer H., Schäfer A., Kiderlen A. F., Masihi K. N., Burger R. (1997). A highly sensitive cytotoxicity assay based on the release of reporter enzymes, from stably transfected cell lines. *Journal of Immunological Methods*.

[18] Von Zons P., Crowley-Nowick P., Friberg D., Bell M., Koldovsky U., Whiteside T. L. (1997). Comparison of europium and chromium release assays: cytotoxicity in healthy individuals and patients with cervical carcinoma. *Clinical and Diagnostic Laboratory Immunology*.

[19] Brasier A. R., Tate J. E., Habener J. F. (1989). Optimized use of the firefly luciferase assay as a reporter gene in mammalian cell lines. *BioTechniques*.

[20] Jacobs W. R., Barletta R. G., Udani R. (1993). Rapid assessment of drug susceptibilities of *Mycobacterium tuberculosis* by means of luciferase reporter phages. *Science*.

[21] Contag C. H., Bachmann M. H. (2002). Advances in in vivo bioluminescence imaging of gene expression. *Annual Review of Biomedical Engineering*.

[22] Brown C. E., Wright C. L., Naranjo A. (2005). Biophotonic cytotoxicity assay for high-throughput screening of cytolytic killing. *Journal of Immunological Methods*.

[23] van Tendeloo V. F. I., Ponsaerts P., Lardon F. (2001). Highly efficient gene delivery by mRNA electroporation in human hematopoietic cells: superiority to lipofection and passive pulsing of mRNA and to electroporation of plasmid cDNA for tumor antigen loading of dendritic cells. *Blood*.

[24] Van Meirvenne S., Straetman L., Heirman C. (2002). Efficient genetic modification of murine dendritic cells by electroporation with mRNA. *Cancer Gene Therapy*.

[25] Mitchell D. A., Nair S. K. (2000). RNA-transfected dendritic cells in cancer immunotherapy. *The Journal of Clinical Investigation*.

[26] Kreiter S., Diken M., Selmi A., Türeci Ö., Sahin U. (2011). Tumor vaccination using messenger RNA: prospects of a future therapy. *Current Opinion in Immunology*.

[27] Holtkamp S., Kreiter S., Selmi A. (2006). Modification of antigen-encoding RNA increases stability, translational efficacy, and T-cell stimulatory capacity of dendritic cells. *Blood*.

[28] Britten C. M., Meyer R. G., Kreer T., Drexler I., Wölfel T., Herr W. (2002). The use of HLA-A^∗^0201-transfected K562 as standard antigen-presenting cells for CD8^+^ T lymphocytes in IFN-*γ* ELISPOT assays. *Journal of Immunological Methods*.

[29] Wölfel T., van Pel A., Brichard V. (1994). Two tyrosinase nonapeptides recognized on HLA-A2 melanomas by autologous cytolytic T lymphocytes. *European Journal of Immunology*.

[30] Skipper J. C. A., Hendrickson R. C., Gulden P. H. (1996). An HLA-A2-restricted tyrosinase antigen on melanoma cells results from posttranslational modification and suggests a novel pathway for processing of membrane proteins. *The Journal of Experimental Medicine*.

[31] Simon P., Omokoko T. A., Breitkreuz A. (2014). Functional TCR retrieval from single antigen-specific human T cells reveals multiple novel epitopes. *Cancer Immunology Research*.

[32] Kreiter S., Selmi A., Diken M. (2008). Increased antigen presentation efficiency by coupling antigens to MHC class I trafficking signals. *Journal of Immunology*.

[33] Jäger E., Nagata Y., Gnjatic S. (2000). Monitoring CD8 T cell responses to NY-ESO-1: correlation of humoral and cellular immune responses. *Proceedings of the National Academy of Sciences of the United States of America*.

[34] Zaritskaya L., Shurin M. R., Sayers T. J., Malyguine A. M. (2010). New flow cytometric assays for monitoring cell-mediated cytotoxicity. *Expert Review of Vaccines*.

[35] Carreno B. M., Magrini V., Becker-Hapak M. (2015). A dendritic cell vaccine increases the breadth and diversity of melanoma neoantigen-specific T cells. *Science*.

[36] Gros A., Robbins P. F., Yao X. (2014). PD-1 identifies the patient-specific CD8^+^ tumor-reactive repertoire infiltrating human tumors. *The Journal of Clinical Investigation*.

[37] Long A. H., Haso W. M., Shern J. F. (2015). 4-1BB costimulation ameliorates T cell exhaustion induced by tonic signaling of chimeric antigen receptors. *Nature Medicine*.

[38] Clynes R., Takechi Y., Moroi Y., Houghton A., Ravetch J. V. (1998). Fc receptors are required in passive and active immunity to melanoma. *Proceedings of the National Academy of Sciences of the United States of America*.

[39] van de Winkel J. G., Anderson C. L. (1991). Biology of human immunoglobulin G Fc receptors. *Journal of leukocyte biology*.

[40] Tuereci O., Woell S., Jacobs S., Mitnacht-Kraus R., Sahin U. (2014). Abstract 2903. IMAB362, a novel first-in-class monoclonal antibody for treatment of pancreatic cancer. *Cancer Research*.

[41] Sahin U., Al-Batran S., Hozaeel W. (2015). IMAB362 plus zoledronic acid (ZA) and interleukin-2 (IL-2) in patients (pts) with advanced gastroesophageal cancer (GEC): clinical activity and safety data from the PILOT phase I trial. *Journal of Clinical Oncology*.

[42] Trarbach T., Schuler M., Zvirbule Z. (2014). Efficacy and safety of multiple doses of IMAB362 in patients with advanced gastro-esophageal cancer: results of a phase II study. *Annals of Oncology*.

[43] Schuler M., Zvirbule Z., Lordick F. (2013). Safety, tolerability, and efficacy of the first-in-class antibody IMAB362 targeting claudin 18.2 in patients with metastatic gastroesophageal adenocarcinomas. *Journal of Clinical Oncology*.

[44] Hoerr I., Obst R., Rammensee H.-G., Jung G. (2000). In vivo application of RNA leads to induction of specific cytotoxic T lymphocytes and antibodies. *European Journal of Immunology*.

[45] Weide B., Carralot J.-P., Reese A. (2008). Results of the first phase I/II clinical vaccination trial with direct injection of mRNA. *Journal of Immunotherapy*.

